# Comparison of arthroscopic single-row and double-row repair in the treatment of rotator cuff tears: A protocol of cohort analysis

**DOI:** 10.1097/MD.0000000000021030

**Published:** 2020-07-17

**Authors:** Yanming Lin, Jiasong Zhao, Heng Qiu, Yong Huang

**Affiliations:** Department of Orthopaedics, Hospital of Chengdu University of Traditional Chinese Medicine, Sichuan, China.

**Keywords:** double-row, protocol, rotator cuff tear, single-row

## Abstract

**Background:**

Single-row (SR) and double-row (DR) techniques are 2 kinds of widely used approaches for the arthroscopic repair of rotator cuff. This retrospective clinical trial was performed to address the question of whether a DR rotator cuff anchor repair gives results superior to a SR anchor repair in clinical outcome scores and complication rates.

**Methods:**

This study was performed and reported in accordance with the Strengthening the Reporting of Observational studies in Epidemiology checklist. We retrospectively reviewed our database, which was collected prospectively. From 2014 to 2017, 264 patients underwent arthroscopic rotator cuff repair by an experienced single shoulder surgeon with the SR and DR techniques. This study was approved by the institutional review board in our hospital and was registered in the Research Registry. Outcome measures included Constant-Murley score, muscle strength, patient satisfaction, passive range of motion, and retear rates.

**Results:**

The hypothesis was that the DR technique would achieve better functional scores and fewer complications as compared to the SR technique in treatment of rotator cuff tears.

## 1 Introduction

For several decades, both open or mini-open techniques, as well as arthroscopic repairs, have exhibited good clinical effects in treating rotator cuff. With constant developments and advances in surgical instrument and technique, open techniques are slowly being replaced by arthroscopic repairs which possess faster recovery and better cosmetic results in rotator cuff repair.^[1,2]^

Single-row (SR) and double-row (DR) techniques are 2 kinds of widely used approaches for the arthroscopic repair of rotator cuff. The SR fixation, which places anchors in a side of the tendon footprint, requires less anchor and suture and demands lower experience degree for surgeon, making a corresponding decrease in cost and operation time compared with the DR repair. On the other hand, several biomechanical studies showed that the use of DR approach incorporated a second row of suture anchors so that increased the contact area tendon to bone. This technique reestablished normal footprint of rotator cuff at the same time. Theoretically, DR fixation can lead to better biomechanical and anatomical outcomes.^[3–7]^ However, improved outcomes in patients who underwent DR approach can hardly been demonstrated in some clinical studies.^[8,9]^ The 2 techniques could both produce acceptable results, so there are still debates about the 2 techniques in the clinical superiority.

Recently, Series of clinical studies reported that the clinical outcomes of arthroscopic SR and DR rotator cuff repair with suture anchor fixation were not different, whereas some reports indicated that DR suture anchor fixation may improve the cuff integrity rate.^[9–14]^ This retrospective clinical trial was performed to address the question of whether a DR rotator cuff anchor repair gives results superior to a SR anchor repair in clinical outcome scores and complication rates. The hypothesis was that the DR technique would achieve better functional scores and fewer complications as compared to the SR technique in treatment of rotator cuff tears.

## 2 Materials and methods

### 2.1 Patients

This study was performed and reported in accordance with the Strengthening the Reporting of Observational studies in Epidemiology checklist. We retrospectively reviewed our database, which was collected prospectively. From 2014 to 2017, 264 patients underwent arthroscopic rotator cuff repair by an experienced single shoulder surgeon with the SR and DR techniques. This retrospective cohort study was approved by the institutional review board in Hospital of Chengdu University of Traditional Chinese Medicine (CUTCM012071) and was registered in the Research Registry (researchregistry5646). The inclusion criteria in this study were

(1) patients with full thickness rotator cuff tear confirmed during arthroscopy,
(2) those with tendon tears that were fully repaired after surgery,
(3) those who were followed up clinically for at least 2 year, and
(4) those who were followed up radiologically for at least 1 year.

Exclusion criteria were

(1) partially repaired tears,
(2) revision cases,
(3) irreparable rotator cuff tears,
(4) partial rotator cuff tears.

### 2.2 Surgical techniques

All operations were performed with the patient in the lateral decubitus position under general anesthesia. Supplemental suprascapular nerve and lateral pectoral nerve blocks were performed on all patients to help control postoperative pain. Hypotensive anesthesia was routinely used, and at no point was any electrocautery device used in the shoulder. The greater tuberosity footprint was debrided of soft tissue and using a shaver blade abraded to create a bleeding surface.

#### 2.2.1 SR technique

Suture anchors were placed on the lateral edge of the greater tuberosity. Metal suture anchors double loaded with No. 2 braided polyester sutures were used in this study. The number of anchors was determined by the tear size: 2 anchors were used for tears measuring 1 to 3 cm, whereas 3 to 4 anchors were required for tears measuring greater than 3 cm. One limb of each suture was passed through the tendon approximately 5 to 10 mm medial to the tear margin and tied in a simple configuration with a sliding knot and backup half-hitches.

#### 2.2.2 DR technique

The medial row of anchors was placed first, at the articular margin of the humeral head, and both limbs of each suture were passed through the tendon part near the muscle-tendon junction of the rotator cuff in a horizontal mattress fashion. For tears measuring 1 to 3 cm, only 1 medial-row anchor was used, and for those greater than 3 cm in size, 2 medial-row anchors were used. The lateral row of anchors was placed in a fashion similar to the SR repair technique: 1 limb of each suture was passed through the tendon approximately 5 to 10 mm medial to the tear margin. Lateral sutures were tied first by use of a sliding knot, and then medial sutures were tied in a mattress configuration with a nonsliding knot. In some cases with large U-shaped or L-shaped tears, the technique combined side-to-side repair with No. 2 braided polyester sutures and tendon-to-bone repair with suture anchors was necessary.

### 2.3 Postoperative rehabilitation

Postoperatively, a shoulder abduction brace was used to immobilize and maintain the shoulder at 30° to 40° internal rotation and 20° abduction. Gentle passive forward flexion began on the operative day. In cases of medium tears, the brace was removed 5 weeks postoperatively, and an active-assisted range of motion (ROM) mobilization, including stick exercise, began. Active resistance muscle strengthening exercises began after 8 weeks using Thera-Band (HCM-Hygienic Corp, Batu Gajah, Malaysia). For large tears, active-assisted ROM and stick exercises began at 6 weeks, and active resistance muscle strengthening exercises at 9 weeks. Three to 4 months postoperatively, patients were allowed to perform light activities. Sports activities and heavy labor were permitted after 9 months.

### 2.4 Outcome evaluation

Outcome measures included Constant-Murley score, muscle strength, patient satisfaction, passive ROM, and retear rates. For muscle strength evaluation, we used a digital dynamometer that measured the maximum strength in pounds after 5 seconds of contraction in the affected and contralateral arm. The mean value of 3 repeated measurements at 90° of elevation in the scapular plane was recorded and also used for scoring strength in the Constant-Murley score. Passive ROM was tested in the following planes using a standard goniometer: flexion, internal rotation and external rotation at 90° of shoulder abduction, and external rotation with arm at side. All patients underwent postoperative magnetic resonance arthrography at the 6-month follow-up and the final follow-up appointment (Table 1).

**Table 1:**
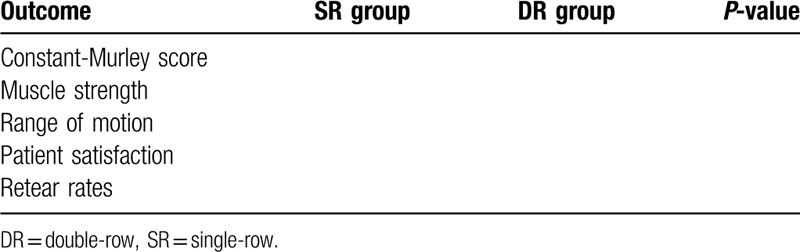
Postoperative outcomes.

### 2.5 Statistical analysis

The Student *t* test and the Mann–Whitney *U* test were used to compare age, duration of symptoms, follow-up period, tear size, ROM, muscle strength, and clinical assessment scores between the 2 groups. Fisher's exact test and the Chi-square test were used to compare gender, affected shoulder, smoker, and retear rate between the 2 groups. The paired *t* test and Wilcoxon sign's rank test were used to compare between the preoperative and postoperative ROM, muscle strength, and constant score. Significance was set at a level of 0.05 with 95% confidence intervals. SPSS software package (version 21.0; SPSS Inc, Chicago, IL) was used for all statistical analyses.

## 3 Discussion

Arthroscopic rotator cuff repair is becoming popular because it has less morbidity than the open technique and yields comparable clinical results. The most common approach to rotator cuff repair using an arthroscopic technique involves the use of suture anchors in either a SR or a DR configuration. The SR repair involves placing anchors either in the lateral aspect of the tendon footprint or lateral to the footprint itself.^[15]^ A DR repair incorporates the same anchor configuration as the SR repair, with the addition of a second row of anchors placed in the medial aspect of the tendon footprint.^[16]^ Studies have demonstrated good clinical outcomes following arthroscopic SR repair. However, techniques have evolved to include a DR in an effort to improve healing rates. A number of basic science studies have compared the 2 techniques and have demonstrated superiority of fixation strength in DR compared with SR repair.^[17,18]^

This retrospective clinical trial was performed to address the question of whether a DR rotator cuff anchor repair gives results superior to a SR anchor repair in clinical outcome scores and complication rates. Limitations of this study included single surgeon practice, single implant manufacturer, and single implant model utilized, and lack of patient randomization. In addition, the limitations of our study also included those inherent in any retrospective cohort study, including the possibility of selection or observational bias.

## Author contributions

**Conceptualization:** Yanming Lin.

**Data curation:** Jiasong Zhao.

**Formal analysis:** Yanming Lin, Jiasong Zhao.

**Funding acquisition:** Yong Huang.

**Investigation:** Yanming Lin, Jiasong Zhao.

**Methodology:** Yanming Lin.

**Resources:** Yong Huang.

**Software:** Heng Qiu.

**Supervision:** Yong Huang.

**Validation:** Heng Qiu.

**Visualization:** Heng Qiu.

**Writing – original draft:** Yanming Lin, Jiasong Zhao

**Writing – review & editing:** Yong Huang.
